# Digital Biomarkers of Social Anxiety Severity: Digital Phenotyping Using Passive Smartphone Sensors

**DOI:** 10.2196/16875

**Published:** 2020-05-29

**Authors:** Nicholas C Jacobson, Berta Summers, Sabine Wilhelm

**Affiliations:** 1 Center for Technology and Behavioral Health Geisel School of Medicine Dartmouth College Lebanon, NH United States; 2 Massachusetts General Hospital Harvard Medical School Boston, MA United States

**Keywords:** biomarkers, machine learning, technology assessment, biomedical, social anxiety, social anxiety disorder, mobile phone

## Abstract

**Background:**

Social anxiety disorder is a highly prevalent and burdensome condition. Persons with social anxiety frequently avoid seeking physician support and rarely receive treatment. Social anxiety symptoms are frequently underreported and underrecognized, creating a barrier to the accurate assessment of these symptoms. Consequently, more research is needed to identify passive biomarkers of social anxiety symptom severity. Digital phenotyping, the use of passive sensor data to inform health care decisions, offers a possible method of addressing this assessment barrier.

**Objective:**

This study aims to determine whether passive sensor data acquired from smartphone data can accurately predict social anxiety symptom severity using a publicly available dataset.

**Methods:**

In this study, participants (n=59) completed self-report assessments of their social anxiety symptom severity, depressive symptom severity, positive affect, and negative affect. Next, participants installed an app, which passively collected data about their movement (accelerometers) and social contact (incoming and outgoing calls and texts) over 2 weeks. Afterward, these passive sensor data were used to form digital biomarkers, which were paired with machine learning models to predict participants’ social anxiety symptom severity.

**Results:**

The results suggested that these passive sensor data could be utilized to accurately predict participants’ social anxiety symptom severity (*r*=0.702 between predicted and observed symptom severity) and demonstrated discriminant validity between depression, negative affect, and positive affect.

**Conclusions:**

These results suggest that smartphone sensor data may be utilized to accurately detect social anxiety symptom severity and discriminate social anxiety symptom severity from depressive symptoms, negative affect, and positive affect.

## Introduction

### Background

Social anxiety disorder (SAD) affects approximately 13% of Americans [1]. It is characterized by an intense, persistent, and exaggerated fear of evaluation or scrutiny in social situations and associated with behavioral avoidance [2]. SAD has a high socioeconomic cost, as it is associated with increased risk of school dropout, reduced productivity in the workplace, and lower quality of life [3]. Individuals with SAD symptoms are also at increased risk for developing depressive disorders [4], with comorbidity rates estimated between 30% and 70% in clinical and community samples [5-7].

Broadly speaking, anxiety around social situations (eg, fear of rejection or embarrassment) is relatively commonplace and can negatively impact individuals even outside the confines of this diagnostic category [8]. Furthermore, given the social inhibition and private anguish inherent to this pathology, SAD symptoms are often mistaken for shyness by others or perceived as a character flaw by the individual. As a result of these misconceptions, the nuanced effects of the condition are frequently underreported and underrecognized. Indeed, individuals with SAD often avoid consulting physicians about their psychological problems [9], and studies estimate that only 35% of individuals who meet clinical criteria receive treatment [3]. Thus, a sizable proportion of individuals who are struggling with SAD symptoms and could benefit from intervention go unaided. Moreover, there is a large time gap between disorder onset and the individual receiving treatment, with 36% experiencing SAD for more than 10 years before seeking help [10]. Currently, much of our understanding of the occurrence and presentation of SAD symptomatology is based on dispositional measures that have limited ecological validity. There is a great need for novel methodologies to improve our understanding and ability to identify individuals who may be vulnerable to developing this debilitating condition.

Smartphones have shown promise in recent years as ecologically valid tools for monitoring and predicting one’s behavior and psychological state [11-14]. Specifically, passive usage and sensor data streams (eg, accelerometers, microphones, and GPS) offer insight into momentary behaviors that can serve as proxies for important mental health variables. The overarching goal of harnessing such data is to better understand, predict, and ultimately intervene when subtle behaviors are suggestive of problematic pathology. These data can be used to evaluate indicators of pathology over time with minimal burden or cost to the individual, researchers, or the broader health care system. Previous research examining patterns of smartphone use by socially anxious individuals have investigated a number of constructs including level of smartphone *addiction* [15,16], communication preferences (eg, preference for texting over voice calls) [17], behavioral markers before outgoing phone calls [18], location data (ie, using GPS) [19], and use of camera and health-related apps [20].

### Previous Findings

Despite the promise of offering a better understanding of the contextual factors related to SAD symptom severity, most of the current research to date has not examined whether symptom severity can be accurately predicted utilizing only sensor data (ie, without additional severity indicators). This investigation is necessary to assess the utility of smartphone sensor data as a stand-alone predictive tool. Particularly, research is needed to determine whether passive sensor data could present a viable alternative to traditional assessments. Some research has examined the within-sample correlations of specific sensor metrics related to SAD symptom severity with significant correlations between some passive sensor data, including location, movement, calls, and texts [19,21]. However, correlations presented in previous publications have been based on general linear models utilizing the entire dataset and, consequently, may overfit the sample and overestimate how well these models would generalize to new independent samples [22]. In addition, the correlations used in these methods were not based on machine learning models, which can combine features to better predict SAD symptom severity (consequently, the absolute correlations between these features and social anxiety were only 0.01-0.36). The study by Boukhechba et al [21] was the only previous study that used the same publicly available data contained within this study.

To date, few researchers have investigated the out-of-sample accuracy of predicting social anxiety from these passive sensors [17,20]. Rather than examining SAD symptom severity continuously, one such study created three categories of SAD symptom severity (low, mid, and high) [18], which artificially and arbitrarily changes the scale and reduces the variance of the outcome [23]. Although previous researchers were successful in predicting social anxiety severity from phone calls, the out-of-sample prediction only explained 15.38% of the total variation in symptoms based on the statistics reported [19], indicating that the majority of the variance is explained by other variables not captured in the model. Consequently, although passive sensor data hold promise in assessing constructs related to SAD symptom severity, more research is needed to determine whether passive data can accurately predict symptoms to the degree that they represent a potential alternative to traditional assessments.

### Study Aims

This study sought to test the utility of passive smartphone sensor data (ie, incoming and outgoing calls, text messages, and accelerometer data), gathered over 2 weeks via the Sensus mobile app as predictors of SAD symptoms in an unselected sample of undergraduates. Studies show that anxiety symptoms are common in undergraduate populations [24], and analog samples are useful for examining this pathology on a continuum [25]. Moreover, the study also utilized this sample to limit the influence of heterogeneity, given that undergraduates often experience similar environmental stressors and life phases. We hypothesized that we would be able to accurately predict SAD symptom severity with at least moderate accuracy (correlations greater or equal to 0.5 between out-of-sample predicted and observed social anxiety symptom severity) [26]. We also hypothesized that the predicted SAD symptom severity would show discriminant validity, evidenced by significantly higher correlations between the observed and predicted SAD symptom severity, compared with correlations with measures of affect (ie, depression symptoms, negative affect, and positive affect). Furthermore, given the novel nature of these data, we had some exploratory aims namely, we were interested in whether the data indicated any nuanced biomarkers that may be worth examining in future iterations of this work.

## Methods

### Participants

The current sample comes from a public use dataset [21]. A total of 72 undergraduate students within the United States consented to participate in the study (37/72, 51% female, mean age 19.8 years, SD 2.4; age range 18-23 years; 30/72, 41% white, 27/72, 37% Asian, 3/72, 4% black, 3/72, 4% Latinx/Hispanic, and 9/72, 12% multiracial or unspecified). Participants were recruited through email advertisements sent to university email listserv for undergraduate students and the psychology department study participant pool. Participants were required to own their own Android devices (with an operating system 4.3 or higher). Although 72 participants were enrolled, only 59 with any phone calls, text data, and accelerometer data across the study period were a part of this study (all three channels were required to be included in the current analysis).

### Measures

#### Social Interaction Anxiety Scale

The Social Interaction Anxiety Scale (SIAS) represented the primary outcome for this study [27]. The SIAS assesses the level of anxiety experienced in social situations using 20 self-report statements. Participants rate each of these items on a 0 (*not at all characteristic of me*) to 4 (*extremely characteristic of me*) Likert scale. In comparing patient populations with nonpatient populations, the SIAS demonstrated superior ability in differentiating SAD from nonanxious controls, with 82% to 86% sensitivity and 82% to 90% specificity using a cutoff point of 34 [28,29]. The SIAS also demonstrated high convergent validity (*r*=0.72) with the social phobia scale [28]. Previous research suggests that the optimal cutoff score for differentiating clinically significant SAD for the SIAS is 36 and that this cutoff point results in 93% sensitivity and 60% specificity in differentiating SAD from other clinically anxious groups, including panic disorder and agoraphobia [30]. Thus, the SIAS has strong convergent and discriminant validity in assessing a range of social anxiety symptoms. Moreover, the internal consistency of the SIAS in the current sample was good (alpha=.83) [11].

#### Depression, Anxiety, and Stress Scale—Depression Scale

The Depression, Anxiety, and Stress Scale (DASS-21) was used to assess depressive symptoms as a measure of discriminant validity for the study. The depression scale has excellent convergent validity (*r*=0.78 with the personal disturbance depression scale, *r*=0.66 with the Hamilton depression scale) [31] and adequate discriminant validity (*r*=0.62 for personal disturbance anxiety scale, *r*=0.59 for the Hamilton anxiety scale) [31]. The depression scale also has excellent internal consistency in the current sample (alpha=.91) [11].

#### Positive Affect Negative Affect Schedule

The positive affect negative affect schedule (PANAS) is a commonly utilized measure of positive and negative affect and was used in this study as a means of assessing discriminant validity [32]. The PANAS is a 20-item instrument that asks participants to rate the degree to which they experience positive (eg, *alert*, *inspired*, *enthusiastic*) and negative (eg, *distressed*, *upset*, *guilty*) affect *in general* on a 1 (*very slightly or not at all*) to 5 (*extremely*) Likert scale. The negative affect subscale of the PANAS has demonstrated convergent validity with the Beck Depression Inventory (*r*=0.58), the Hopkins Symptom Checklist (*r*=0.74), and the State Trait Anxiety Inventory state anxiety scale (A-State; *r*=0.51); while the positive affect subscale is negatively correlated with these measures (*rs*=−.35, −.19, and −.35, respectively [32]). Both subscales exhibit good internal consistency (alpha negative affect=.87, alpha positive affect=.88 [32]; note that the raw items were not reported for this sample, so the internal consistency of these scales is unknown for the current sample).

#### Passive Sensor Data

Accelerometer data were collected once per second (1/Hz) for the duration of the study. Notably, accelerometers sampled at this frequency have been used to detect related psychopathology (eg, major depression and bipolar disorders; pain and worry severity) [33-35]. Likewise, incoming and outgoing calls and text timestamps were recorded during the study period (note that the text message content was not included). Passive sensor data were collected for approximately 2 weeks (mean 16.41 days, SD 2.69).

### Procedure

The study was approved by the institutional review board at the University of Virginia. Participants received partial course credit or payment for their participation, and they were told that the study examined how their thoughts and feelings interacted with their daily environment. They were also instructed on the type of data that would be collected from their mobile phones. Individuals provided informed consent and attended two laboratory visits. During the first laboratory visit, they completed the SIAS and other measures (not relevant to this study). After this, participants installed the Sensus mobile app onto their own Android phones. They returned approximately 2 weeks later, where they completed additional measures and were debriefed (although the authors may have collected these same measures during the follow-up assessment, only the baseline assessments were released).

### Planned Analyses

#### Overview of Analyses

Before any analyses, a set of biomarkers was created for each person’s data. Note that passive sensor data are based on within-person variation (ie, changes over time), and yet the primary hypothesis is based on a between-person question (ie, stable individual differences across people, which matches previous research conceptualizing anxiety disorders as dynamical systems) [36]. Consequently, the same set of biomarkers was extracted separately for each person from the time series of their passive sensor data, and, then, these biomarkers were used for interindividual analyses.

#### Accelerometer Biomarkers

The accelerometer data were processed consistently with a previously published procedure [33]. The first feature creation was used to describe the overall distribution of the outcome scores; this included the following raw data metrics: (1) the mean, (2) median, (3) mode, (4) minimum, (5) maximum, (6) skewness, (7) kurtosis, and (8) SD. Furthermore, we included (9) the root mean square of successive difference from 1 and 2 lags difference, and (10) the first through 99th quantiles in increments of 1 percentile. Thus, these features were predominantly created to extract relevant signals related to the functional form of the raw data.

The second set of features was created to represent the autoregressive dynamic nature of the raw data, which can measure complex relationships of temporal stability and oscillatory patterns while imposing a constraint for smoothness. This was constructed using the differential time-varying effect model, which is based on a generalized additive modeling framework utilizing the following formula for each person. In particular, the mean of *y_i,l’_*, *μ_i_*≡*E*(*y_i,l’_*) is linked to a semiparametric predictor, *η_i_*, expressed as:

*η_i_*=*f*_1_(*TD_i,l_*)*y_i,l_*

Here, *y* represents the raw data for each measurement *i*, at lag *l*. Note that *l’* represents a stacked vector of the outcome such that the same time series is stacked repeatedly to account for each potential lagged relationship *l*. The term *f*_1_ represents a smooth based on a thin-plate regression spline. Note that the term *f*_1_ is a nonparametric component wherein the effects of a series of covariates on the mean of the transformed dependent response variable are of unknown functional forms. TD reflects the time difference between the measurement occasion *i* and lag *l*. The primary term interest is thus: *f*_1_(*TD_i,l_*)*y_i,l_*, which is a varying-coefficient model representing the linear relationship between the lagged outcome of *y* on itself at later time points as a function of nonlinear time differences [37]. Features were created separately for each person’s raw data (ie, one model per person). The extracted feature was the predicted varying-coefficients across the entire time series from all possible lags.

The third set of features was based on the spectral analysis, wherein each of the estimated power spectral densities was recovered for the raw data [38]. This represents the decomposed cyclical patterns that are common throughout the time series within the data. The power density describes the degree of strength of the variation in the raw data as a function of frequency. There was no missing accelerometer biomarker data.

#### Text Message Biomarkers

Notably, the text message data were much sparser than the accelerometer data, and consequently, fewer biomarkers could be derived. The distributional outcomes described within the accelerometer biomarkers above (eg, mean, median, mode, etc) were applied to the vector of the time difference between the text messages with the goal of processing the distribution of the time difference between adjacent text messages (attempting to capture whether the participant engaged in text messages with long delays or shorter-time periods). This was first applied for all text messages, then all incoming text messages, and, finally, all outgoing text messages. The typical distribution variance between persons was also captured by taking the same distributional features of the time differences for each person that the person contacted, and then calculating the SD of the distribution of time differences (note that this was to account for the potential that a socially anxious person may vary in the speed with which they text back persons with whom they are close as opposed to persons with whom they enjoy a more distant relationship). This resulted in a measure of how much variation there was in the length of time between contacts. Finally, the last feature that was extracted was the number of total texts. There was no missing text message biomarker data.

#### Call Biomarkers

The following features were created to process the call data: (1) the total number of calls, (2) the percentage of calls that were missed, (3) the percentage of calls that came during times the phone was idle (ie, to capture a time in which they might have been interrupted from their current tasks, which could facilitate avoidance among those with high social anxiety), (4) the number of total persons that the participants contacted, and (5) the distribution features extracted from the time differences between calls (ie., similar to the text messages, this approach was to account for how long it would take a person to call someone back, which we suspected could be higher in persons with high social anxiety). There was no missing call biomarker data.

#### Machine Learning

After each of the features were created, an ensemble of extreme gradient boosting machines (*XGboost*) was utilized. Extreme gradient boosting machines are learning algorithms composed of several weak tree-based learners. They have shown to be more robust at predicting outcomes compared with many traditional algorithms and are often the algorithm that consequently wins machine learning competitions for this reason. The lower level extreme gradient boosting learners were blocked in the following way: (1) predicting social anxiety from the accelerometer distribution features (which constituted one model), (2) predicting social anxiety from the accelerometer autoregressive dynamics (which comprised a second model), (3) predicting social anxiety from the accelerometer spectral densities (trained based on spectral densities in blocks of 1000 features each), (4) predicting social anxiety from the text message biomarkers, and (5) predicting social anxiety from the call biomarkers. Note that each of the extreme gradient–boosting models utilized leave-one-out cross-validation, such that the features that were extracted were the predictions from the model without including that participant in the model (ie, all features were out-of-sample predictions). Finally, the final ensemble model was trained based on the model predictions of the lower order models, with the higher-order model also being based on an extreme gradient boosting model. The final ensemble model also utilized leave-one-out cross-validation, such that the final model was based on an out-of-sample prediction.

#### Outcome Metrics

The primary outcome of interest was the correlation between the predicted and observed SAD symptom severity scores. In addition, the discriminant validity of the predicted SAD symptom scores was also compared by comparing the correlation between the SAD symptom scores based on the smartphone biomarkers and depression, negative affect, and positive affect. As there was missing data in the depression (5% missing), negative affect (14% missing), and positive affect (14% missing), multiple imputation was utilized to estimate the correlations for the discriminant validity. Similar to Ortiz et al [39], discriminant validity was compared by comparing the correlations between predicted SAD symptom severity based on the smartphone biomarkers and the observed SAD symptom severity strength, compared with the correlation between the smartphone biomarkers and depression, negative affect, and positive affect, respectively [40].

The variable importance from the model ensemble was identified, such that the most important feature will be extracted and plotted to determine the most important contribution to the model predictions. In addition, the t-distributed stochastic neighbor embedding (t-SNE) plot was examined from the lower order ensemble models and the final predictions to visualize the degree of separation between the predictors and social anxiety symptoms. The t-SNE plot is a visual depiction of how well the machine learning models naturally separate different degrees of SAD symptom severity. The variable importance of the primary feature was also extracted from the higher-order ensemble, as well as from the lower order model to determine the single most influential digital biomarker of the predictions of SAD symptom severity.

## Results

### Percentage of the Sample With Clinical Anxiety

**Figure 1:**
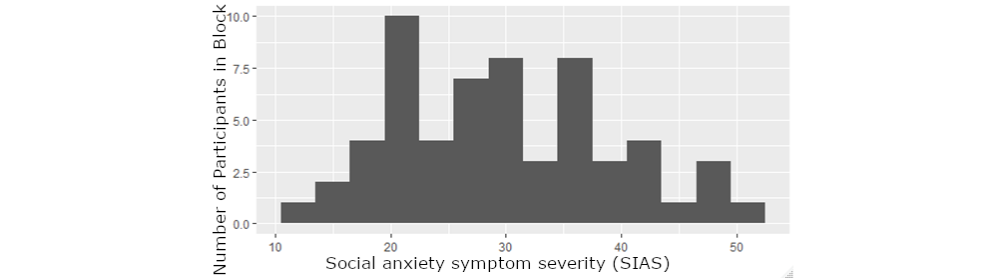
Social anxiety symptom severity based on the social interaction anxiety scale.

The distribution of the SAD symptom severity is depicted in Figure 1. The mean of the SIAS was 29.125, with an SD of 9.407, and a range from 11 to 52. On the basis of previous validated cutoffs (score of 34 on the SIAS), 36% (21/59) of the sample was above the primary cutoff, suggesting they were at clinical levels of SAD. In applying the more conservative cutoff of 36 used in clinical outpatient samples to differentiate anxiety from other anxiety disorders, a total of 34% (21/59) of the sample was very likely to have clinically significant social anxiety (and not merely other anxiety disorders, based on the results of a previous study suggesting that this clinical cutoff discriminated SAD from other anxiety disorders [30]).

### Primary Results

#### Convergent Validity

**Figure 2:**
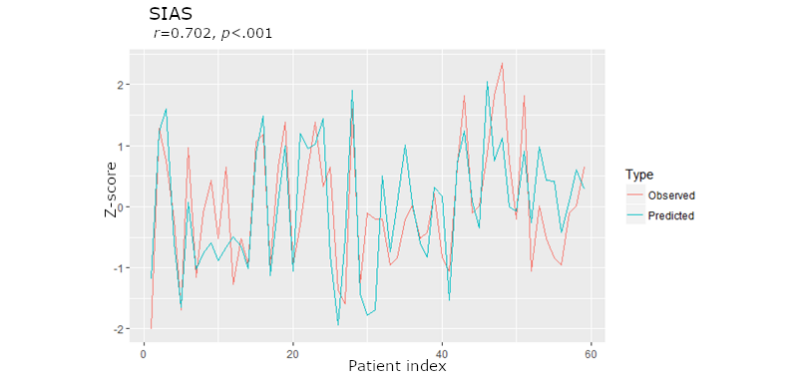
Z-scores of the predicted social anxiety disorder (SAD) severity and the observed SAD symptom severity for each participant. Note that the patient index represents each of the 59 participants and not a continuous metric. SAD: social anxiety disorder; SIAS: social interaction anxiety scale.

**Figure 3:**
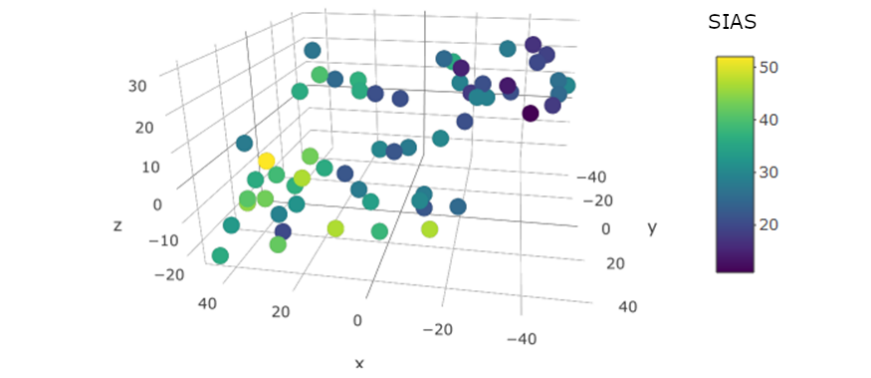
t-distributed stochastic neighbor embedding (t-SNE) plot depicting the ensembled model features (ie, the predictions extracted from the lower order ensembles) and the observed social anxiety symptoms. Note that closer points in the t-SNE are shown to be similar to one another, and dissimilar objects are shown to be farther away from one another. Note that the axes themselves are used to reduce the dimensionality of the machine learning features, so the Dimension 1, Dimension 2, and Dimension 3 labels are not of interest. This plot shows the ability to differentiate the level of social anxiety symptom severity based on the ensembled model features. As can be seen, the combination of the lower order ensembles was able to well differentiate SAD symptom severity. SAD: social anxiety disorder; SIAS: social interaction anxiety scale; t-SNE: t-distributed stochastic neighbor embedding.

The results suggested that there was a strong correlation between predicted and observed social anxiety symptom severity *r*=0.702, 95% CI 0.543-0.812; *P*<.001 (see Figure 2 for a plot of the individual level predictions). Supporting our hypothesis, the bounds of the 95% CI suggested that the strength of the correlation was above 0.5. See Figure 3 for a visual depiction of the degree of separation of the lower order ensemble model features and SAD symptom severity.

#### Discriminant Validity

Regarding the discriminant validity of the measure, the results suggested that there was a weaker but positive relationship between the predicted SAD symptom severity from smartphone biomarkers and depression severity (*r*=0.357, 95% CI 0.112-0.562; *P*=.005), and this correlation is significantly lower than that between the predicted SAD symptom severity from the smartphone biomarkers and the observed SAD symptom severity (*Z*=3.441; *P*<.001). Likewise, the predicted SAD symptom severity from smartphone biomarkers had a positive correlation with negative affect (*r*=0.384, 95% CI 0.143-0.583; *P*=.003), and the correlation is significantly lower than that between the predicted and observed SAD symptom severity (*Z*=3.484; *P*<.001). The predicted SAD symptoms had a nonsignificant negative correlation with positive affect (*r*=−0.138, 95% CI −0.380 to 0.122; *P*=.32), which was significantly lower than the correlation between predicted and observed SAD symptom severity (*Z*=5.980; *P*<.001). The magnitude of the variance explained was also more than three times greater between the predicted and observed SAD severity, compared with the variance explained by the predicted SAD severity and each of the discriminant constructs. This suggests that smartphone biomarkers demonstrate discriminant validity between depression, negative affect, and positive affect. Note that the partial correlation between the predicted SAD severity and the observed SAD severity was also still strong and significant when controlling for depression, positive affect, and negative affect (*r*=0.502, 95% CI 0.283 to 0.671; *P*<.001).

#### Exploratory Aims

**Figure 4:**
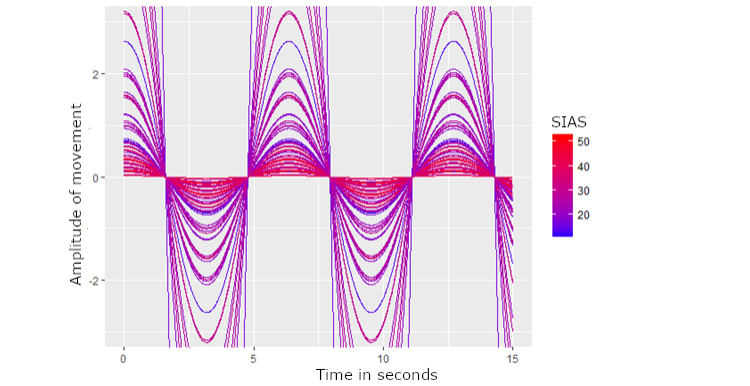
Oscillations of movement at approximately every 6 seconds and the relationship to social anxiety symptom severity. Note that each line represents a separate person. The lines are shaded from blue (low social anxiety symptoms) to red (high social anxiety symptoms). This graph clearly depicts that those with high social anxiety symptoms tended to have lower amplitudes of these 6-second oscillations than those with higher social anxiety symptoms. SIAS: social interaction anxiety scale.

Note that based on the variable importance metrics, the oscillations occurring every 6.35 seconds appeared to be the most influential contributor to the final ensemble model (see Figure 4). Graphical depictions of the results showed clear differences in amplitude between those with higher social anxiety symptoms and those with lower social anxiety symptom severity, such that those with low social anxiety tended to have greater oscillatory frequency patterns during this 6-second timespan.

## Discussion

### Principal Findings

Our data indicated that calls, texts, and movement patterns captured by individuals’ smartphones over 2 weeks provided sufficient information to predict severity of SAD symptoms with moderately strong accuracy (*r*=0.702), and that these patterns can also accurately discriminate social anxiety from depression (*r*=0.357), negative affect (*r*=0.384), or positive affect (*r*=−0.138). Although preliminary, these data are promising, as they suggest that simple behavioral information that is already passively collected for most individuals in the United States may represent a highly feasible, low-cost, low-burden, and specific mechanism for identifying people who are vulnerable to experiencing problematic levels of social anxiety.

Note that the present findings provide unique and incremental contributions of the previous public use dataset [21]. In particular, the previous research trained models based on the entire dataset and did not use a holdout sample, which may result in overfitting the sample and overestimate how well these models would generalize to new independent samples [22]. In addition, the correlations used via these methods were not based on machine learning models, which may combine features to better predict SAD symptom severity (consequently, the absolute correlations between these features and social anxiety were only 0.01-0.36) [21]. By utilizing a combination of features, rather than single features, the correlations were substantially better within the current sample despite being based on out-of-sample predictions.

The current findings are particularly notable when one considers the concurrent validity of SAD symptom measures. In particular, the convergent validity of these predictions is approximately equivalent to the convergent validity between established social anxiety symptom scales (eg, *r*=0.730 between the SIAS and the Liebowitz Social Anxiety Scale) [41]. This suggests that the accuracy of these behavioral phenotypes is approximately equivalent to the validity of symptom measures, which is particularly noteworthy given that self-report scales are known to tend to overly correlate with one another more strongly than other methods due to the artifact of shared method variance alone [42]. This has important implications for the field of psychology, as behavioral profiles are not based on subjective feelings, but rather replicable observable phenomena. Although this work is preliminary and more work is needed to develop a broader constellation of digital biomarkers, these behavioral profiles may be interesting outcomes to help organize and conceptualize psychiatric disorders themselves.

This study has many notable strengths. In particular, the smartphone app collected passive data from participants over 2 weeks continuously (once per second), allowing for rich behavioral signals that are well beyond the temporal precision available in existing social anxiety measures. Likewise, this study also applied some cutting-edge machine learning techniques in analyzing these digital biomarkers. This study also utilized leave-one-out cross-validation to examine the overall performance, which directly examines the degree to which the trained model generalizes to unseen data, and these models continued to suggest that they had high predictive accuracy. Finally, this study included a large percentage of those at clinical levels of SAD.

### Limitations

This study has some limitations that provide direction for future research. First, our sample size was modest, and participants were all undergraduate students, which may limit the generalizability of findings as SAD affects individuals of all ages and educational backgrounds. Furthermore, although 36% (20/59) of the sample reported symptom severity above the suggested clinical cutoff on the SIAS, we did not use a clinical sample in this study. Thus, it will be important that future replications of this work be conducted in larger, more representative samples. Larger samples would also allow for more power to test possible moderating variables (eg, gender). Second, passive data collection does not allow us to quantify the type of social contact that someone may be experiencing (ie, it may be difficult to infer whether someone is contacting a relative or a distant acquaintance). Third, the current models were trained to predict between-person differences, and, consequently, it is unknown whether the results would generalize to predicting within-person variability.

Our data also revealed that the most influential contributor to the predictive model was oscillatory frequency patterns during a 6.35-second timespan, such that less socially anxious individuals evidenced greater oscillatory frequency than high-social anxiety counterparts. Given the nature of the speed, we suspect that this reflects the length of several sequential strides during walking (ie, where this pace would reflect a slightly below average stride of six consecutive strides based on persons average walking pace) [43]. We suspect that this oscillatory pattern occurred as it best reflected continued walking frequency (ie, between 1 and 2 strides in a local environment likely has very high instability as persons only navigate very little distance). Nevertheless, given the novel nature of this metric, the true implications of this finding are unclear. It is possible that this pattern reflects persons with low social anxiety walking at a consistent confident and steady pace, whereas persons with high social anxiety walking might walk more quickly and less confidently or at an irregular pace. Continued research is needed to clarify the degree to which this finding is specific to this population, and the relevance of this metric for our understanding of symptom presentation.

### Conclusions

Taken together, our study extends recent efforts to utilize passive smartphone sensor data to improve the field’s ability to detect nuanced behavioral indicators of problematic pathology [44,45]. This is especially important for individuals with social anxiety, given that the occurrence of these symptoms is more frequent than is typically reported. However, this method of harnessing naturally occurring behavioral data is certainly relevant for identifying and better understanding a range of maladaptive thoughts and behaviors that underlie psychiatric conditions more broadly.

## Abbreviations

DASS-21: Depression, Anxiety, and Stress Scale
PANAS: positive affect negative affect schedule
SAD: social anxiety disorder
SIAS: social interaction anxiety scale
t-SNE: t-distributed stochastic neighbor embedding
